# Multi-Omics Characterization of Genome-Wide Abnormal DNA Methylation Reveals FGF5 as a Diagnosis of Nasopharyngeal Carcinoma Recurrence After Radiotherapy

**DOI:** 10.3390/biom15020283

**Published:** 2025-02-14

**Authors:** Zhi-Qing Long, Ran Ding, Ting-Qiu Quan, Rui Xu, Zhuo-Hui Huang, Denghui Wei, Wei-Hong Zheng, Ying Sun

**Affiliations:** 1 Department of Radiology, Sun Yat-sen University Cancer Center, State Key Laboratory of Oncology in South China, Collaborative Innovation Center of Cancer Medicine, Guangdong Key Laboratory of Nasopharyngeal Carcinoma Diagnosis and Therapy, Guangdong Provincial Clinical Research Center for Cancer, Guangzhou 510060, China; longzq@sysucc.org.cn (Z.-Q.L.);; 2 Department of Radiation Oncology, Sun Yat-sen University Cancer Center, State Key Laboratory of Oncology in South China, Guangdong Key Laboratory of Nasopharyngeal Carcinoma Diagnosis and Therapy, Guangdong Provincial Clinical Research Center for Cancer, Guangzhou 510060, China; quantq@sysucc.org.cn (T.-Q.Q.); weidh@sysucc.org.cn (D.W.)

**Keywords:** multi-omics characterization, nasopharyngeal carcinoma, FGF5

## Abstract

Background: Aberrant expression and mutations in the fibroblast growth factor (FGF) family play crucial roles in cell differentiation, growth, and migration, contributing to tumor progression across various cancers. Nasopharyngeal carcinoma (NPC), a malignancy prevalent in East Asia, is primarily treated with radiotherapy; however, radioresistance remains a major challenge, leading to recurrence and poor outcomes. While FGFs are known to activate signaling pathways such as MAPK, PI3K/AKT, and JAK/STAT to promote cancer progression, the specific role of individual FGFs in NPC radioresistance remains unclear. Emerging evidence highlights *FGF5* as a key player in NPC progression, metastasis, and radioresistance, underscoring its potential as a therapeutic target to overcome treatment resistance and improve clinical outcomes. Methods: We analyzed single nucleotide variation (SNV) data, gene expression, and DNA methylation patterns using cancer datasets, including TCGA and GTEx, to investigate *FGF5* expression. Differentially expressed genes (DEGs) were identified and interpreted using functional enrichment analysis, while survival analysis and gene set enrichment analysis (GSEA) were conducted to identify clinical correlations. DNA methylation patterns were specifically assessed using the HumanMethylation850 BeadChips on tissue samples from nine recurrent and nine non-recurrent NPC patients. Functional assays, including cell viability, migration, invasion, and clonogenic survival assays, were performed to evaluate the effects of FGF5 on NPC cell behavior in vitro and in vivo. Results: *FGF5* showed elevated SNV frequencies across multiple cancers, particularly in HNSC and NPC. DNA methylation analysis revealed an inverse relationship between *FGF5* expression and methylation levels in recurrent NPC tumors. Functional assays demonstrated that FGF5 enhances migration, invasion, and radioresistance in NPC cells. High FGF5 expression was associated with reduced distant metastasis-free survival (DMFS) and increased radioresistance, highlighting its role in metastatic progression and recurrence. Conclusions: FGF5 plays a significant role in the progression and recurrence of nasopharyngeal carcinoma. Its elevated expression correlates with increased migration, invasion, and radioresistance as well as reduced distant metastasis-free survival. These findings suggest that FGF5 contributes to the metastatic and recurrence potential of NPC, making it a potential target for therapeutic intervention in treating these cancers.

## 1. Introduction

Nasopharyngeal carcinoma (NPC) is a unique subset of head and neck cancers, characterized by its distinct geographical distribution and etiology. The incidence of NPC is significantly higher in Southern China, Southeast Asia, and North Africa, with 3.0 per 100,000 in China to 0.4 per 100,000 in populations that are mainly white [1,2]. NPC is typically classified by histological subtypes—keratinizing squamous cell carcinoma, non-keratinizing differentiated carcinoma, non-keratinizing undifferentiated carcinoma, and other rarer forms. Despite these histopathological differences, clinical management is driven primarily by disease staging rather than histological classification [3].

Radiotherapy, often combined with chemotherapy, remains the cornerstone of treatment for non-metastatic NPC, with generally favorable outcomes [4]. However, NPC has a pronounced tendency for distant metastasis, even among head and neck cancers. This is especially true for recurrent or metastatic NPC (RM-NPC), where treatment options are limited and prognosis is worse. Approximately 15% of patients present with distant metastasis at diagnosis, and an additional 20% of those with locoregionally advanced disease experience metastasis following initial treatment [5]. For these patients, therapeutic strategies are typically restricted to palliative systemic therapies, which are often associated with limited efficacy and significant toxicity.

NPC is considered radiosensitive, with platinum-based chemotherapy regimens yielding response rates of over 80% in first-line treatment for metastatic cases [1]. Nevertheless, the emergence of resistance to radiotherapy continues to pose a significant challenge, highlighting the urgent need for innovative therapeutic strategies. Additionally, the molecular mechanisms driving NPC progression and metastasis are still not fully understood. Epithelial-to-mesenchymal transition (EMT) [6], a critical process in which epithelial cells acquire mesenchymal characteristics to enhance metastatic potential, has been implicated in NPC, as in many other cancers. EMT is orchestrated by key transcription factors, such as Snail, Twist, and ZEB1/2, which mediate the downregulation of epithelial markers like E-cadherin and the upregulation of mesenchymal markers. Despite advances in understanding EMT’s role in cancer progression, significant gaps remain in elucidating the molecular mechanisms that facilitate NPC metastasis and therapeutic resistance [7].

Recent studies have highlighted the role of cancer-associated fibroblasts (CAFs) in regulating chemotherapy sensitivity by modulating ferroptosis, a form of iron-dependent cell death. Ref. [7] demonstrated that cisplatin (DDP) induces ferroptosis in NPC cells [7], but CAF-secreted fibroblast growth factor 5 (*FGF5*) inhibits this process by activating the FGFR2/Keap1/Nrf2/HO-1 pathway, thereby promoting chemoresistance. Targeting the FGF5/FGFR2 axis offers a promising avenue to enhance DDP sensitivity and overcome resistance in RM-NPC [8].

In our study, we performed methylation sequencing on tumor samples from NPC patients who had undergone radiotherapy, identifying significant upregulation of *FGF5* gene expression in those with distant metastasis. We further explored the role of *FGF5* in promoting NPC’s malignant phenotypes and analyzed its clinical relevance to patient prognosis. These findings provide new insights into the molecular mechanisms underlying NPC metastasis and radioresistance, potentially guiding the development of novel therapeutic strategies aimed at improving patient outcomes.

## 2. Material and Methods

### 2.1. Study Subjects and Biospecimens

Tissue samples were obtained from the Sun Yat-sen University Cancer Center (SYSUCC; Guangzhou, China), including 9 paraffin-embedded specimens from patients who experienced tumor recurrence within 5 years after radiotherapy as well as 9 samples from patients without recurrence (Human ethics approval code: No. B2022-313-01 Date: 18 May 2022). The demographic characteristics and clinical data of the participants were retrieved from medical records. The study protocol was approved by the Institutional Ethical Review Board of Sun Yat-sen University Cancer Center, and all patients provided written informed consent upon admission for the use of their tissue samples. All procedures, including the use of human tissue specimens and analysis of clinical data, were conducted in strict accordance with the guidelines of the Declaration of Helsinki.

### 2.2. Cell Lines and Cell Culture

All cell lines used in this study were authenticated and kindly provided by Dr. M. Zeng (Sun Yat-Sen University Cancer Center) [9,10,11,12,13]. Human nasopharyngeal carcinoma (NPC) cell lines were cultured in Roswell Park Memorial Institute (RPMI)−1640 medium (Invitrogen, Carlsbad, CA, USA), both supplemented with 10% fetal bovine serum (FBS; Invitrogen, AUSTRILIA). The human immortalized nasopharyngeal epithelial cell line NP69 was maintained in keratinocyte serum-free medium (KSFM, Invitrogen, USA), supplemented with bovine pituitary extract (BD Biosciences, San Diego, CA, USA).

### 2.3. Quantitative Real-Time PCR Analysis

Total RNA was extracted from cells using TRIzol reagent (Invitrogen, USA), and cDNA was synthesized with the HiScript III RT SuperMix (Vazyme, Nanjing, China) following the manufacturer’s protocol. Quantitative real-time PCR (qPCR) was performed using a CFX96 Touch Real-Time PCR System (Bio-Rad, Hercules, CA, USA) with SYBR Green qPCR SuperMix-UDG reagents (Invitrogen, AU). The threshold cycle (Cq) values were determined in triplicate for each sample. GAPDH (glyceraldehyde-3-phosphate dehydrogenase) was used as the internal control for normalization. The primer sequences for qPCR analysis were as follows: *FGF5*: Forward: GGAATACGAGGAGTTTTCAGCAAC, Reverse: CTCCCTGAACTTGCAGTCATCTG. *GAPDH*: Forward: GTCTCCTCTGACTTCAACAGCG, Reverse: ACCACCCTGTTGCTGTAGCCAA.

### 2.4. Plasmid Construction and Transfection

The coding region of *FGF5* was tagged with FLAG and cloned into empty vector plasmids to generate the overexpression construct pCDH-EF1-Flag-FGF5-T2A-puro. For transient transfections, cells were transfected with the overexpression plasmids or small interfering RNAs (siRNAs) using Lipofectamine 3000 (Invitrogen) following the manufacturer’s protocols. Cells were harvested 24–48 h post-transfection for further analysis. The sequences of siRNAs used in this study are as follows: Negative control: 5′- CCUACAUCCCGAUCGAUGAUGUUGA-3′; *FGF5*-si1: 5′-GCCCUAUCAAGCCAAAGAUTT-3′; FGF5-si2: 5′-GAUCCCACGAAGCCAAUAUTT-3′.

### 2.5. DNA Extraction and Methylation Data Processing

Genomic DNA was isolated from tissue samples using the Allprep DNA/RNA Kit (Qiagen, GERMANY) and sequenced using the Infinium HumanMethylation850 BeadChips (850 K array, Illumina) to assess genome-wide methylation patterns. Data preprocessing, normalization, and β-value calculation were performed using the R package minfi (version 1.26.2) [14]. Gene annotation was classified into six regions: TSS1500, TSS200, 5′UTR, 1st Exon, gene body, and 3′UTR. CpG island annotation was categorized into five regions: N shelf, N shore, CpG Island, S shore, and S shelf (see figure below).

We applied the following quality control criteria: (i) probes with detection *p*-values ≥ 0.01 in more than 5% of the samples were removed; (ii) probes located on the X or Y chromosomes were excluded; (iii) probes overlapping single nucleotide polymorphisms (SNPs) were eliminated; (iv) probes mapped to multiple loci in the human genome were discarded. After applying these criteria, 866,092 probes were retained for further analysis.

The CpG probe annotation file was downloaded from the ENCODE Project database (http://genome.ucsc.edu/ENCODE/downloads.html, accessed on 15 November 2024.) [15]. Each CpG probe was annotated with the corresponding gene, genomic region (TSS1500: 200–1500 bases upstream of the transcriptional start site [TSS]; TSS200: 0–200 bases upstream of the TSS; 5′UTR: within the 5′ untranslated region between the TSS and the ATG start codon; gene body: between the ATG start codon and the stop codon, irrespective of the presence of introns or exons; 3′UTR: between the stop codon and the poly A signal), CpG island-associated regions (shore: 0–2 kb from the island; shelf: 2–4 kb from the island; N: upstream of the 5′ end of the CpG island; S: downstream of the 3′ end of the CpG island), and functional regions (enhancer: predicted enhancer elements in the original 850 K design marked as “true”; DHS: DNase I hypersensitivity sites).

### 2.6. Identification of Differentially Methylated CpG Sites

We employed the IMA package in R to identify differentially methylated CpG sites and regions between sample groups. The analysis was performed based on both gene annotation categories and CpG island annotation categories. For each region, the signal values of all probes were averaged, and differential methylation regions (DMRs) were identified using the pooled t-test method [16]. The selection criteria for DMRs were set at *p* < 0.05 and |Beta.Difference| > 0.14. The Beta value (β), which ranges between 0 and 1, represents the methylation level at a given site, where values closer to 1 indicate higher methylation and values closer to 0 indicate lower methylation.

The formula for calculating the Beta value (β) is as follows:

$${beta}_{i}=\frac{max(y_{\left(i,methy\right)},0)}{\max\left(y_{\left(i,methy\right)},0\right)+\max\left(y_{\left(i,unmethy\right)},0\right)+100}$$

In this study, the Beta value (β) was used to compare differential methylation between sample groups. The Beta value density curves of the samples were generated to illustrate the overall methylation distribution of all CpG sites within each sample, allowing for visualization of potential differences in global methylation patterns across samples.

### 2.7. Cell Viability and Migration or Invasion Assays

Cell Proliferation Assay: Cell proliferation was assessed using the Cell Counting Kit-8 (CCK-8; Dojindo Laboratories, Kumamoto, Japan). A total of 1 × 10^3^ cells per well were seeded in a 96-well plate, and cell viability was measured every 24 h for 5 days following the manufacturer’s instructions.

Transwell Migration and Invasion Assays: For the migration and invasion assays, 5 × 10⁴ cells (for migration) or 1 × 10⁵ cells (for invasion) were seeded into the upper chambers of Transwell plates (BD Biosciences, San Jose, CA, USA) in FBS-free medium. For the invasion assays, the chambers were coated with Matrigel (BD Biosciences). The lower chambers were filled with medium containing 10% FBS. After 24 h of incubation, the cells that migrated or invaded to the lower surface of the membrane were fixed, stained with hematoxylin, and examined under an inverted microscope.

Clonogenic Assay: For clonogenic survival, single-cell suspensions (200–10,000 cells per well) were seeded into 6-well plates and treated with ionizing radiation (IR) at doses ranging from 0 to 6 Gy. After 10–14 days, once colonies had formed, the plates were washed with PBS, fixed in methanol, and stained with crystal violet. Colonies containing more than 50 cells were counted. Survival fractions (SF) were calculated using the linear-quadratic model: SF = exp (−αD − βD^2^), where D is the radiation dose.

### 2.8. Identification of Differential Methylation Expression and Gene Set Enrichment Analysis

To identify DMGs, we performed comparisons between groups of patients with recurrence and non-recurrence in NPC as well as between samples with high and low *FGF5* expression. For both groups, we applied a Student’s *t*-test on log2-transformed gene expression levels, quantified by transcripts per million (TPM). Genes with an FDR-adjusted *q*-value < 0.05 and a mean expression level > 1 or < 0.50 (tumor vs. normal or high vs. low *FGF5* expression) were considered significantly different. To further validate these results, we used the Wilcoxon signed-rank test, which confirmed that 99.75% of genes remained differentially expressed (*p*-value < 0.05, relative fold change > 1) across both comparisons, including all genes used in downstream analyses. Functional enrichment analysis of the identified DEGs was conducted using the clusterProfiler package (version 4.0.5) [17]. Gene ontology (GO) enrichment analysis focused on the “biological process” category, using both the enrichmentGO and GSEA functions in clusterProfiler. The false discovery rate (FDR) was adjusted using the Benjamini–Hochberg method to account for multiple comparisons.

### 2.9. Data Download and Processing

We analyzed 31 solid tumor types from The Cancer Genome Atlas (TCGA), including ACC, BLCA, BRCA, CESC, CHOL, COAD, ESCA, GBM, HNSC, KICH, KIRC, KIRP, LGG, LIHC, LUAD, LUSC, MESO, OV, PAAD, PCPG, PRAD, READ, SARC, SKCM, STAD, TGCT, THCA, THYM, UCEC, UCS, and UVM. The full names of these tumor types and sample sizes are provided in Supplementary Table S1 of the Supporting Information. Normal tissue data were sourced from the Genotype-Tissue Expression (GTEx) database (https://gtexportal.org/home, 10 February 2025) [18]. Somatic SNV data for these 31 tumor types were obtained from the GSCA database (http://bioinfo.life.hust.edu.cn/GSCA/#/, 10 February 2025) [19], while RNA-seq gene expression data were retrieved from the UCSC Xena platform (https://xenabrowser.net/datapages/, 10 February 2025) [20].

### 2.10. Murine Xenograft Model of Nasopharyngeal Carcinoma: Tumor Growth and Irradiation Study

The animal experimental procedures were approved by the Institutional Animal Care and Use Committee of Sun Yat-sen University Cancer Center (No. GZR2022-224). Murine xenograft growth of nasopharyngeal carcinoma (NPC) was studied using female BALB/c nude mice (4–6 weeks old, n = 10), purchased from Charles River Laboratories (Beijing, China). The mice were housed in a barrier facility under a 12 h light/dark cycle at 18–22 °C with 50–60% humidity. They were randomly assigned to two groups for subcutaneous tumor injections: one group received 1 × 10⁶ 5-8F-shNC cells (negative control), and the other group received 5-8F-*shFGF5* cells with *FGF5* knockdown. Tumor volume and body weight were measured every three days, starting on day seven post-injection. Once the xenograft tumors reached approximately 5 mm in diameter, the mice were subjected to a single 10 Gy local irradiation. Tumor volume was calculated using the formula length × width^2^ × 0.5. All experimental procedures were approved by the Institutional Animal Care and Use Committee of Sun Yat-sen University Cancer Center and adhered to the guidelines of the Declaration of Helsinki. Efforts were made to minimize animal suffering. The maximal allowable tumor diameter of 20 mm, as permitted by the ethics committee, was not exceeded in this study (Animal ethics approval code: No. GZR2022-224 Date: 3 March 2022).

### 2.11. Statistical Analysis

Kaplan–Meier survival analysis and log-rank tests were performed using the R package survival (version 3.2-13) to assess overall survival [21]. All statistical analyses, including Student’s *t*-test, Wilcoxon rank-sum test, and ANOVA, were carried out in R (version 4.2.0) [22]. Statistical significance was defined as *p* < 0.05. Data visualization, including bar charts, pie charts, line graphs, and other figures, was generated using the ggplot2 R package (version 3.4.2) [23], while heatmaps were constructed with the ComplexHeatmap R package (version 2.14.0) [24].

## 3. Results

### 3.1. Genomic Landscape of the FGF Gene Family in Pan-Cancer and Head and Neck Cancer

**Figure 1:**
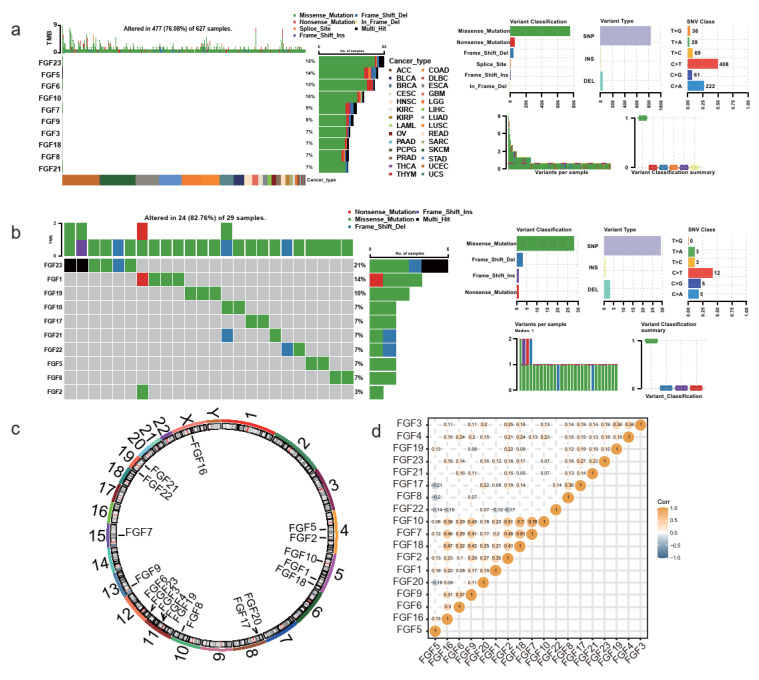
Genomic alterations and expression landscape of the FGF gene family in pan-cancer and head and neck cancer. (**a**) Oncoplot showing the single nucleotide variant profile of the FGF gene family across pan-cancer. (**b**) Summary of SNV classes in the FGF gene family. (**c**) Chromosomal locations of the FGF gene family members. (**d**) Correlation heatmap of FGF gene family expression across cancer types.

To provide a comprehensive understanding of the FGF gene family in cancer, we analyzed SNVand expression patterns across multiple cancer types. Notably, *FGF5* and *FGF23* exhibited the highest mutation frequencies among the FGF gene family, with SNV frequencies ranging from 10% to 68% in tumors such as UCEC, SKCM, READ, ESCA, COAD, STAD, ACC, BLCA, LUSC, and LGG (Figure 1a). Among 627 examined samples, 477 (76.08%) harbored mutations in at least one FGF gene, with particularly high mutation burdens observed in UCEC and SKCM.

In head and neck cancer, the mutation frequency of FGF genes was relatively uniform, ranging from 7% to 14%, with FGF23 showing a distinct mutation profile (Figure 1b). Chromosomal distribution and mRNA expression correlation analysis further revealed that FGF family members share high expression correlations, suggesting coordinated regulatory mechanisms (Figure 1c,d).

These findings highlight the significant variation in mutation rates among cancer subtypes and underscore the potential oncogenic roles of *FGF5* and *FGF23* in multiple tumors. The high mutation burden in UCEC and SKCM suggests that these cancers may be particularly susceptible to FGF family alterations, identifying these pathways as promising therapeutic targets. In contrast, the relatively consistent mutation frequencies in head and neck cancer suggest that FGF genes, with the exception of *FGF23*, may play more conservative roles in tumor progression.

### 3.2. Genome-Wide DNA Methylation Analysis Identifies FGF5 as a Key Regulator in NPC Recurrence

**Figure 2:**
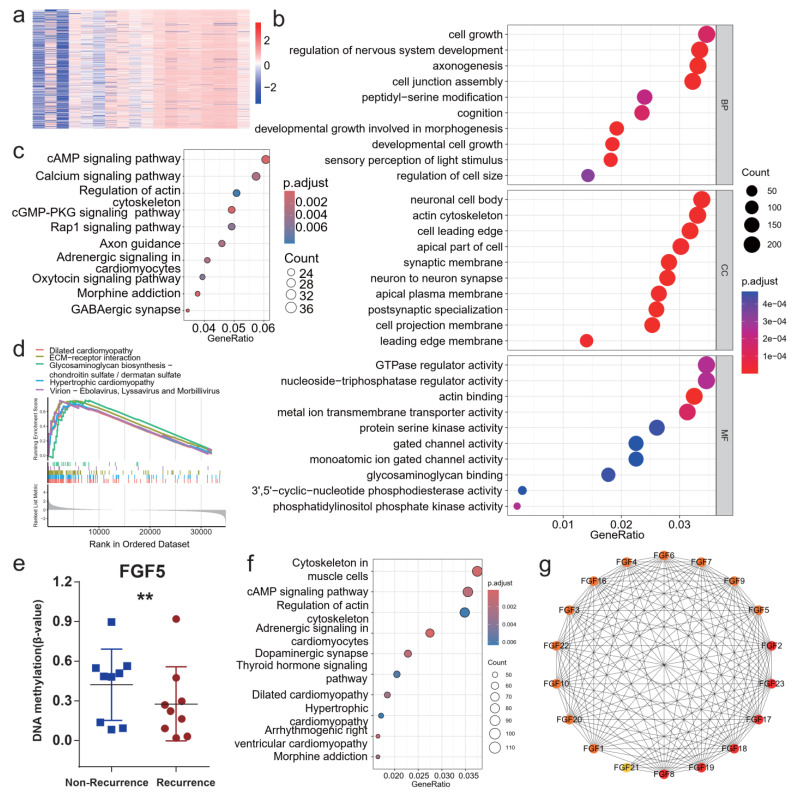
Epigenetic regulation of *FGF5* and its role in NPC recurrence post-radiotherapy. (**a**) Heatmap of methylation signal intensity in NPC patient samples. (**b**,**c**) Biological processes (BP), cellular components (CC), and molecular functions (MF) (**b**) and KEGG pathway (**c**) enrichment analysis of differentially methylated genes in NPC samples. (**d**) Gene set enrichment analysis (GSEA) of differentially methylated genes in NPC samples. (**e**) Boxplot comparing *FGF5* methylation signals between recurrence and non-recurrence NPC samples. (**f**) KEGG enrichment analysis of genes associated with high or low *FGF5* methylation signals. (**g**) Protein–protein interaction network of the FGF gene family; **, *p* < 0.01.

To explore the underlying relationship between methylation and recurrence following radiotherapy in NPC, we conducted a differential methylation analysis comparing recurrence and non-recurrence patient groups, selecting genes with a *p*-value < 0.01 and |Δβ| > 0.2. Table 1 describes the clinical characteristics of these patients in NPC. A total of 844 differentially methylated genes (DMGs) were identified, including 611 genes with downregulated methylation in non-recurrent patients and 233 genes with upregulated methylation in recurrent patients (Figure 2a). Functional enrichment analysis revealed that DMGs were significantly involved in biological processes such as cell junction assembly, axonogenesis, and nervous system development as well as pathways regulating the actin cytoskeleton and intracellular signaling (Figure 2b,c). Gene set enrichment analysis (GSEA) revealed that patients with recurrent tumors had enrichment in pathways such as dilated cardiomyopathy, ECM-receptor interaction, glycosaminoglycan biosynthesis—chondroitin sulfate/dermatan sulfate, and hypertrophic cardiomyopathy as well as viral interaction pathways involving Ebolavirus, Lyssavirus, and Morbillivirus (Figure 2d).

Notably, *FGF5* exhibited significantly lower methylation levels in recurrent patients compared to non-recurrent patients (Figure 2e). Pathway enrichment analysis of *FGF5*-associated genes highlighted critical pathways, including actin cytoskeleton regulation and cAMP signaling, which are known to promote cell migration and survival (Figure 2f). Protein–protein interaction network analysis further supported the central role of FGF5 in regulating cellular processes related to tumor recurrence (Figure 2g).

These findings suggest that DNA methylation of *FGF5* is a key epigenetic regulator in NPC recurrence after radiotherapy. The consistent identification of *FGF5*-associated pathways underscores its potential as a diagnostic marker and therapeutic target for mitigating tumor recurrence.

### 3.3. FGF5 Expression Is Regulated by DNA Methylation and Associated with NPC Progression

**Figure 3:**
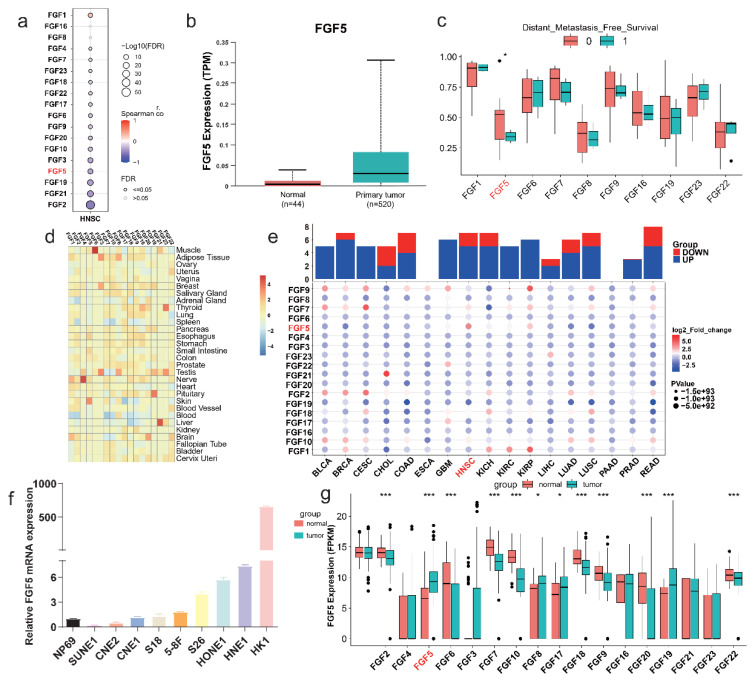
Clinical relevance and functional analysis of FGF5 in NPC. (**a**) Correlation profile between methylation levels and mRNA expression of FGF genes. (**b**) Boxplot comparing TPM values of *FGF5* in TCGA head and neck cancer samples and adjacent normal tissues. (**c**) Boxplot comparing methylation signals of the FGF gene family between patients with and without distant metastasis. (**d**) Heatmap of FGF gene family expression across normal tissues from the GTEx database. (**e**) Pan-cancer expression heatmap of the FGF gene family in TCGA datasets, with red indicating upregulation and blue indicating downregulation. (**f**) RT-PCR analysis of *FGF5* mRNA expression in NP69 (normal nasopharyngeal epithelial) and NPC cell lines. (**g**) Boxplots comparing FGF gene family expression between TCGA head and neck cancer samples and normal tissues; ***, *p* < 0.001, *, *p* < 0.05.

To explore the relationship between *FGF5* expression and DNA methylation, we analyzed head and neck squamous cell carcinoma (HNSC) data from TCGA. Most FGF family members exhibited an inverse correlation between methylation and expression levels, except for *FGF16* and *FGF8*, while *FGF1* showed a positive correlation (Figure 3a). Notably, *FGF5* expression was significantly higher in tumor samples compared to normal tissues, whereas its methylation levels were markedly reduced in NPC patients with distant metastases (Figure 3b,c). To further examine FGF gene expression across different human tissues, we utilized GTEx data. Our analysis revealed distinct tissue-specific expression patterns, with *FGF5* primarily expressed in skin tissue, *FGF6* primarily expressed in muscle tissue, *FGF23* primarily expressed in thyroid tissue, and *FGF4* primarily expressed in nerve tissue (Figure 3d). These findings suggest that FGF genes have highly specialized functions depending on tissue type, playing critical roles in both normal physiological processes and pathological conditions, particularly in cancer. Focusing on HNSC tumors, we observed a significant overexpression of *FGF5* compared to normal tissues (Figure 3e). Consistently, *in vitro* experiments confirmed higher FGF5 expression in NPC cell lines compared to the immortalized nasopharyngeal epithelial cell line NP69, with HK1 and HNE-1 cells displaying the highest expression levels (Figure 3f), Transcriptome data from public databases also showed the same expression trend (Figure 3g).

These results highlight DNA methylation as a key regulatory mechanism of FGF5 expression in NPC, where its dysregulation may contribute to tumor progression and recurrence. The epigenetic regulation and overexpression of *FGF5* in NPC tumors underscore its potential as a diagnostic biomarker and therapeutic target for NPC recurrence.

### 3.4. FGF5 Promotes Tumor Invasion and Metastasis in NPC

**Figure 4:**
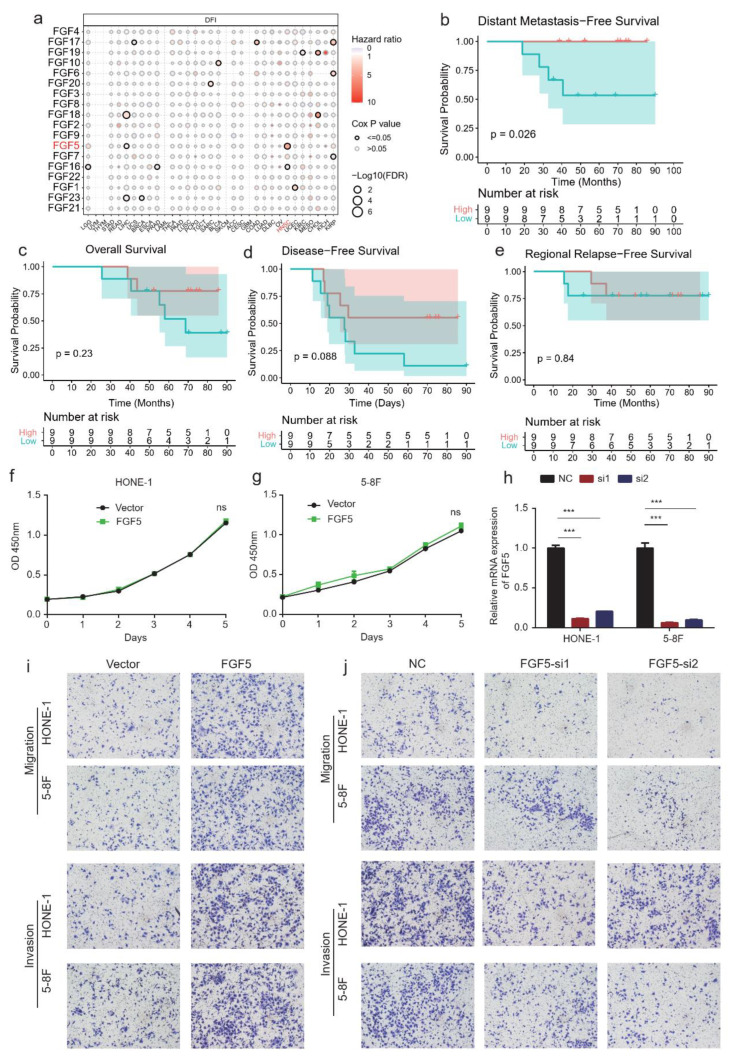
Prognostic value and functional impact of FGF5 in NPC progression and metastasis. (**a**) Bubble heatmap showing the correlation between FGF gene family expression and disease-free interval (DFI) in the TCGA pan-cancer cohort. (**b**–**e**) Kaplan–Meier survival analysis of distant metastasis-free survival (DMFS), overall survival (OS), disease-free survival (DFS), and relapse-free survival (RRFS) in NPC patients stratified by high or low *FGF5* expression. (**f**–**h**) CCK-8 assays evaluating the proliferation ability of HONE-1 (**f**) and 5-8F (**g**) cells transfected with vector or *FGF5*-overexpression plasmid and relative mRNA expression of *FGF5* (**h**). (**i**) Transwell assays assessing migration and invasion capacity of 5-8F and HONE-1 cells transfected with empty vector or *FGF5* overexpression plasmid. (**j**) Transwell assays evaluating migration and invasion capacity of 5-8F and HONE-1 cells transfected with scrambled control or si-FGF5; ***, *p* < 0.001, ns, non-significant.

To assess the functional role of FGF5 in NPC progression, we performed survival analysis and functional experiments. Pan-cancer survival analysis revealed that increased *FGF5* expression was associated with a higher hazard ratio (HR) for disease-free interval (DFI) in HNSC patients (Figure 4a). In NPC patients, lower *FGF5* methylation levels were significantly associated with distant metastasis-free survival (DMFS) but not with overall survival (OS), disease-free survival (DFS), or regional relapse-free survival (RRFS) (Figure 4b–e).

Functional experiments demonstrated that FGF5 knockdown significantly impaired the migratory and invasive capabilities of NPC cells, while exogenous overexpression enhanced these capacities (Figure 4f–j). These results suggest that FGF5 plays a critical role in promoting cancer cell invasion and metastasis, particularly in the context of NPC recurrence.

FGF5 contributes to NPC progression by promoting tumor cell invasion and metastasis, and its methylation status may serve as a prognostic marker for distant metastasis in NPC patients.

### 3.5. FGF5 Modulates Radiation Response and Enhances Radioresistance in NPC

**Figure 5:**
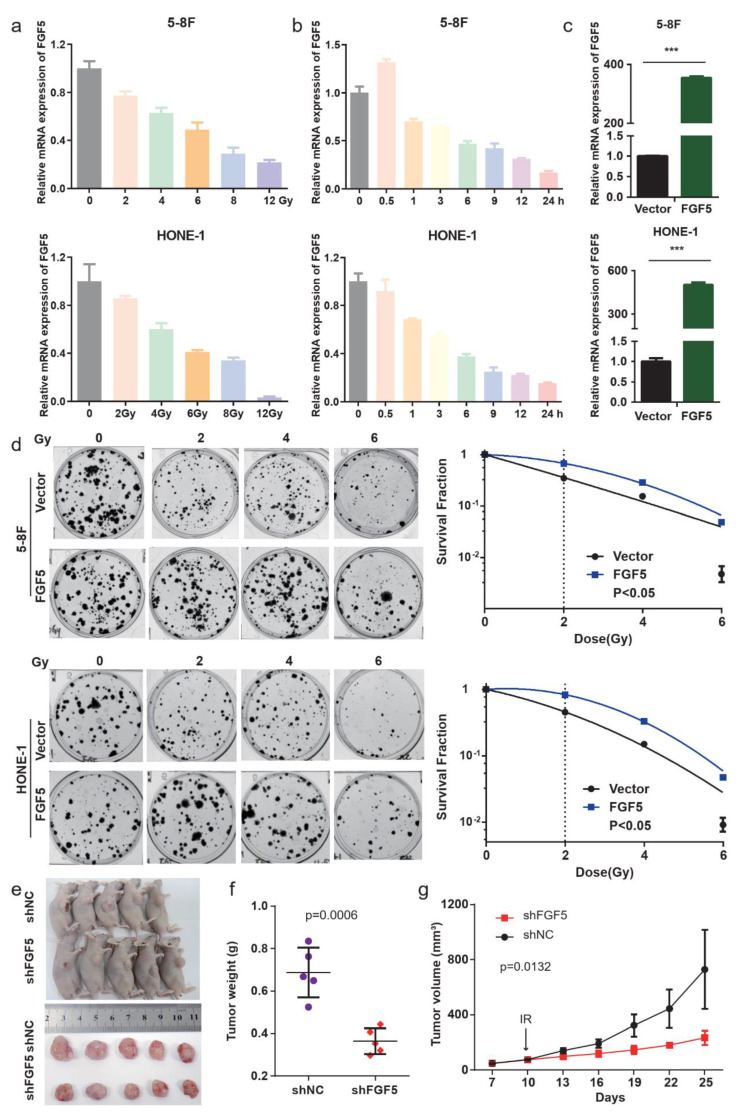
FGF5 enhances radioresistance and modulates radiation response in NPC. (**a**) qPCR analysis of *FGF5* mRNA expression in NPC cell lines 6 h after irradiation (IR) at indicated doses. *GAPDH* was used as a loading control. (**b**) qPCR analysis of *FGF5* mRNA expression at indicated time points after 6 Gy irradiation. *GAPDH* was used as a loading control. (**c**) Validation of *FGF5* overexpression in HONE-1 and 5-8F NPC cell lines using qRT-PCR. *GAPDH* was used as an endogenous control. (**d**) Clonogenic assays and survival fraction curves of SUNE1 and HONE1 cells stably transfected with FGF5 or empty vector plasmids after exposure to indicated irradiation doses. (**e**–**g**) In vivo tumor growth analysis: (**e**) Subcutaneous xenograft tumors retrieved from mice on day 25 after inoculation, (**f**) tumor weight measurements, and (**g**) tumor volume measurements over 25 days in xenografts with or without FGF5 depletion; ***, *p* < 0.001.

To investigate the role of FGF5 in radiation response, we treated NPC cell lines with ionizing radiation (IR). FGF5 expression progressively decreased with increasing radiation doses, suggesting its sensitivity to radiation-induced DNA damage (Figure 5a,b). However, stable FGF5-overexpressing cell lines demonstrated enhanced proliferation and survival following IR exposure, indicating a role in promoting radioresistance (Figure 5c,d).

In vivo experiments further confirmed that FGF5 downregulation enhanced radiosensitivity, as evidenced by reduced tumor growth in xenograft models (Figure 5e–g). These findings highlight the dual role of FGF5 in NPC, where its expression is downregulated in response to radiation yet its overexpression provides a survival advantage.

FGF5 modulates radiation response in NPC, and its downregulation enhances radiosensitivity, suggesting its potential as a therapeutic target to improve radiotherapy outcomes.

## 4. Discussion

Nasopharyngeal carcinoma (NPC) is a malignancy that exhibits significant geographical variation, with the highest incidence found in East and Southeast Asia [25]. While recent advances in treatment have led to improvements in outcomes for most NPC patients, those with locally advanced stages (Stage III-IVa according to the 8th AJCC TNM Staging Classification) continue to experience recurrence following radiotherapy. Understanding the molecular mechanisms underlying this treatment resistance is critical for developing more effective therapeutic strategies [26].

Our investigation into fibroblast growth factor 5 (FGF5) reveals its pivotal role in NPC, particularly regarding treatment resistance and recurrence after radiotherapy [7]. The samples analyzed in our methylation sequencing study were derived from patients who had experienced recurrence following radiotherapy, providing a unique opportunity to assess the molecular landscape associated with treatment failure [27,28]. Notably, we observed that *FGF5* expression was significantly elevated in these recurrent NPC samples compared to those from patients who did not recur. This finding suggests that FGF5 may be intricately involved in the processes leading to treatment resistance, potentially through its regulation of the tumor microenvironment and interaction with cancer-associated fibroblasts (CAFs) [29].

Methylation analysis revealed an inverse relationship between *FGF5* expression and DNA methylation levels. In recurrent NPC samples, reduced methylation at the FGF5 locus correlated with increased gene expression, reinforcing the notion that epigenetic regulation plays a crucial role in the persistence and progression of NPC post-radiotherapy [30]. The hypomethylation of *FGF5* may facilitate its overexpression, contributing to the tumor’s aggressive behavior and ability to evade treatment-induced apoptosis. This underscores FGF5 as a potential biomarker for identifying patients at higher risk of recurrence after radiotherapy.

Survival analyses further highlighted the significant association between *FGF5* expression levels and disease-free intervals (DFI) in NPC patients [31,32]. Patients with higher *FGF5* expression exhibited shorter DFIs, indicating that *FGF5* not only serves as a marker of recurrence risk but may also reflect the tumor’s inherent aggressive characteristics. This correlation emphasizes the need for further investigation into FGF5 as a prognostic marker that could guide treatment decisions and surveillance strategies in NPC management [33].

However, recent studies have highlighted that CAFs, which are more resistant to radiation and chemotherapy, may serve as an important source of FGF5 in the tumor microenvironment [7,34]. Even if tumor-cell-derived FGF5 could be downregulated by cisplatin or radiation, CAFs could continue to produce FGF5, thereby promoting tumor cell survival and migration. This potential compensatory mechanism underscores the importance of targeting both tumor cells and the tumor microenvironment, particularly CAFs, to fully overcome treatment resistance.

In conclusion, our study elucidates the critical role of FGF5 in the recurrence of NPC following radiotherapy, with its expression patterns closely linked to both methylation changes and patient survival outcomes. The distinct expression of FGF5 in recurrent NPC samples along with its regulatory relationship with DNA methylation identify FGF5 as a key player in the pathogenesis of NPC and a promising target for therapeutic intervention. Future research should focus on validating FGF5 as a biomarker for recurrence risk and exploring targeted therapies that could enhance treatment efficacy and improve patient prognosis. Additionally, the role of CAFs as a potential source of FGF5 in the tumor microenvironment warrants further investigation, as targeting CAF-derived FGF5 may provide a novel strategy to overcome treatment resistance in NPC.

## 5. Limitations of This Study

One limitation of our study is the relatively small sample size in our cohort, which may affect the generality of our findings regarding the role of FGF5 in nasopharyngeal carcinoma (NPC). While we were able to replicate our results in other independent cohorts, a larger sample size is needed to validate FGF5 as a reliable biomarker of relapse, especially in the context of radiation response. In addition, while our methylation sequencing data provided insight into *FGF5* expression in methylation and its potential significance in tumor progression and treatment resistance, the limited number of samples from patients who relapsed after radiotherapy limited our ability to draw definitive conclusions about the association between *FGF5* expression, methylation changes, and clinical outcomes. Expanding our patient cohort is critical to assess the impact of confounders, such as inflammation and other comorbidities, on FGF5 expression and its potential role as a therapeutic target for nasopharyngeal cancer. In addition, further studies of the specificity and sensitivity of FGF5 in combination with current therapeutic modalities are critical to fully elucidate its therapeutic potential to overcome therapeutic resistance.

## 6. Conclusions

In this study, we employed a multi-omics approach to investigate the role of FGF5 in NPC recurrence following radiotherapy. Our findings demonstrate that *FGF5* is a key epigenetic regulator in NPC, with its expression closely associated with DNA methylation changes and patient survival outcomes. Reduced methylation at the FGF5 locus in recurrent NPC samples correlated with increased gene expression, suggesting that epigenetic dysregulation of FGF5 contributes to treatment resistance and tumor progression. Functional experiments revealed that FGF5 promotes tumor cellmigration, and invasion, while its downregulation enhances radiosensitivity in NPC. Notably, we observed that FGF5 expression in tumor cells is downregulated after irradiation treatment, yet its overexpression or re-expression significantly enhances tumor cell migration, highlighting its complex involvement in NPC biology and treatment resistance. The integration of DNA methylation and gene expression data underscores the importance of FGF5-associated pathways, such as actin cytoskeleton regulation and cAMP signaling, in driving tumor recurrence. Furthermore, we propose that cancer-associated fibroblasts (CAFs), which are more resistant to radiation and chemotherapy, may serve as a compensatory source of FGF5 in the tumor microenvironment, sustaining tumor cell survival and migration under treatment stress. Our study identifies FGF5 as a promising diagnostic biomarker for NPC recurrence risk and a potential therapeutic target, providing new insights into the molecular mechanisms underlying NPC recurrence and treatment resistance. Targeting FGF5 and its associated pathways, including CAF-derived FGF5, may offer novel therapeutic strategies to mitigate recurrence and improve outcomes for NPC patients. Future research should focus on validating FGF5 as a prognostic marker and exploring targeted therapies that disrupt FGF5 signaling in both tumor cells and the tumor microenvironment.

## Figures and Tables

**Table 1:** Clinical characteristics of patients between LRFS = 0 and LRFS = 1 in NPC.

Variables		Total	LRFS = 0	LRFS = 1
Sex	male	10	5	5
	female	8	4	4
ALB (g/L)	range	40–48.6	43.4–47.3	40–48.6
CRP (mg/L)	range	0.18–4.77	0.97–4.77	0.18–4.03
LDH (U/L)	range	133–231	148–231	133–221.2
Age	range	31–69	31–66	35–69
Family History	yes	7	3	4
	no	11	6	5
T stage	1	1	1	0
	2	5	3	2
	3	12	5	7
N stage	0	1	0	1
	1	12	8	4
	2	4	1	3
	3	1	0	1
OS	alive	11	7	4
	death	7	2	5
DMFS	yes	4	2	2
	no	14	7	7
RRFS	yes	4	0	4
	no	14	9	5
LRRFS	yes	9	0	9
	no	9	9	0
DFS	yes	12	3	9
	no	6	6	0

ALB: Albumin; CRP: C-reaction protein; LDH: Lactate dehydrogenase; OS: Overall survival; RRFS: Regional relapse-free survival; DMFS: Distant metastasis-free survival; DFS: Disease-free survival.

## Data Availability

The human and animal study protocol was approved by the Institutional Ethical Review Board of Sun Yat-sen University Cancer Centel (Human ethics approval code: No. B2022-313-01 Date: 18 May 2022, Animal ethics approval code: No. GZR2022-224 Date: 3 March 2022). Thirty-one kinds of tumor types of somatic SNV and CNV data are publicly accessible at GSCA: Gene Set Cancer Analysis web site (http://bioinfo.life.hust.edu.cn/GSCA/, 1 January 2024.). Gene expression RNAseq data for pan-cancer samples were obtained from the UCSC Xena database (https://xenabrowser.net/datapages/, 17 March 2024.). GTEx: The Genotype-Tissue Expression (https://gtexportal.org/home/, 5 January 2024.). Methylation sequencing raw data are in supplement material.

## Supplementary Materials

The following supporting information can be downloaded at: https://www.mdpi.com/article/10.3390/biom15020283/s1, Supplementary Table S1. Cancer types analyzed in this study and their abbreviations, Supplementary Table S2. Results of differential methylation gene analysis.
