# Supplementary material for: Multi-Omics Characterization of Genome-Wide Abnormal DNA Methylation Reveals FGF5 as a Diagnosis of Nasopharyngeal Carcinoma Recurrence After Radiotherapy
Source: Biomolecules. 2025 Feb 14;15(2):283. doi: 10.3390/biom15020283 (PMC11853517; doi:10.3390/biom15020283)
Supplement: Supplementary file 1 [file biomolecules-15-00283-s001.zip › biomolecules-3452951-Supplementary-Tables.pdf]

SUPPLEMENTARY TABLES

Supplementary Table S1. Cancer types analyzed in this study and their abbreviations.

Cancer type	Abbreviation	Sample number
Adrenocortical carcinoma	ACC	79
Bladder urothelial carcinoma	BLCA	408
Breast invasive carcinoma	BRCA	1102
Cervical squamous cell carcinoma and endocervical adenocarcinoma	CESC	306
Cholangiocarcinoma	CHOL	36
Colon adenocarcinoma	COAD	451
Esophageal carcinoma	ESCA	185
Glioblastoma	GBM	166
Head and neck squamous cell carcinoma	HNSC	522
Kidney chromophobe	KICH	66
Kidney renal clear cell carcinoma	KIRC	534
Kidney renal papillary cell carcinoma	KIRP	291
Lower grade glioma	LGG	529
Liver hepatocellular carcinoma	LIHC	373
Lung adenocarcinoma	LUAD	517
Lung squamous cell carcinoma	LUSC	501
Mesothelioma	MESO	87
Ovarian serous cystadenocarcinoma	OV	308
Pancreatic adenocarcinoma	PAAD	179
Pheochromocytoma and paraganglioma	PCPG	184
Prostate adenocarcinoma	PRAD	498
Rectum adenocarcinoma	READ	160
Sarcoma	SARC	263
Skin cutaneous melanoma	SKCM	472
Stomach adenocarcinoma	STAD	415
Testicular germ cell tumors	TGCT	139
Thyroid carcinoma	THCA	513
Thymoma	THYM	120
Uterine corpus endometrial carcinoma	UCEC	533
Uterine carcinosarcoma	UCS	57
Uveal melanoma	UVM	80

Supplementary Table S2. Results of differential methylation gene analysis.

Gene	Diff_Beat	P_Value	Change
ZWILCH	-0.2478883	0.0030623	DOWN
ZSWIM6	-0.2105121	0.00621601	DOWN
ZSCAN29	-0.2221693	0.00621601	DOWN

ZSCAN2	-0.2117745	0.00621601	DOWN
ZRANB2-AS2	-0.2200028	0.00854701	DOWN
ZNRF3	0.26674302	0.00854701	up
ZNF823	-0.2648364	0.00086841	DOWN
ZNF726	-0.204161	0.00438777	DOWN
ZNF672	0.25678708	0.0000457	up
ZNF669	-0.2219594	0.0030623	DOWN
ZNF662	-0.2006009	0.00621601	DOWN
ZNF639	-0.229794	0.00854701	DOWN
ZNF512	-0.2341512	0.00438777	DOWN
ZNF484	-0.2033646	0.00086841	DOWN
ZNF451	-0.2290682	0.00438777	DOWN
ZNF423	0.2191521	0.00438777	up
ZNF391	0.22048258	0.0030623	up
ZNF366	-0.2221622	0.0030623	DOWN
ZNF202	0.23158574	0.0000914	up
ZIC3	0.31988861	0.00854701	up
ZIC2	0.3412165	0.00854701	up
ZFP64	0.25393258	0.0030623	up
ZDHHC22	0.21754114	0.00054847	up
ZDHHC19	-0.2227077	0.00438777	DOWN
ZC3HAV1L	0.23273254	0.0030623	up
ZBTB38	0.28523848	0.0030623	up
ZBTB12	-0.2036569	0.00854701	DOWN
Z83851.3	-0.2007839	0.00054847	DOWN
YIPF5	-0.2058085	0.00438777	DOWN
YARS2	-0.2405527	0.00438777	DOWN
XXbac-BPG55C20.7	-0.2404218	0.00031994	DOWN
XXbac-BPG308K3.6	-0.2075694	0.00438777	DOWN
XXbac-B461K10.4	-0.206585	0.0030623	DOWN
WNT9A	0.21058366	0.00621601	up
WI2-81516E3.1	0.28249658	0.00438777	up
WHAMMP2	-0.2083762	0.0030623	DOWN
WFS1	-0.2357026	0.00621601	DOWN
WDR69	-0.2261002	0.00438777	DOWN
WDR45	-0.2275455	0.0000914	DOWN
WDR13	0.24088677	0.00031994	up
VWA3B	0.21243898	0.0000457	up
VGLL2	0.27048683	0.00854701	up
USP51	-0.2140301	0.00438777	DOWN
UQCRB	-0.2066955	0.0030623	DOWN
UMODL1	-0.2206041	0.00438777	DOWN
UGT2B4	0.20648418	0.00621601	up
U8	0.20279114	0.00086841	up

TTC39B	-0.2197863	0.00621601	DOWN
TSR2	-0.207084	0.00854701	DOWN
TSPAN2	-0.2005672	0.00438777	DOWN
TSHZ3	0.23318567	0.0030623	up
TRPV6	-0.2237408	0.00854701	DOWN
TRIM6	0.29828999	0.00854701	up
TRIM23	-0.2032931	0.0030623	DOWN
TRBV6-1	0.2039247	0.00854701	up
TRBV23-1	0.27982996	0.00854701	up
TRAPPC10	0.27313979	0.0030623	up
TOP2A	-0.2328914	0.00621601	DOWN
TNMD	-0.2006761	0.00438777	DOWN
TMTC1	0.23151242	0.00137118	up
TMSB15A	-0.2225943	0.00854701	DOWN
TMEM72-AS1	0.23851319	0.00137118	up
TMEM163	0.20042611	0.0030623	up
TMEM140	0.22022343	0.00438777	up
TJP1	-0.2399182	0.00621601	DOWN
TFE3	-0.2479445	0.00621601	DOWN
TFDP3	-0.2660457	0.00854701	DOWN
TCERG1L	0.20815585	0.00438777	up
TATDN1	-0.220776	0.00438777	DOWN
TAS2R5	-0.2515951	0.00205677	DOWN
SYTL2	0.22238378	0.00438777	up
SYT2	0.20564677	0.0030623	up
SYNPO	0.23202173	0.00438777	up
SYNM	-0.2181894	0.00438777	DOWN
SYDE2	-0.2260775	0.00854701	DOWN
SYCP3	-0.2055466	0.00086841	DOWN
STYXL1	0.24867264	0.00438777	up
STON2	0.22555007	0.00137118	up
STIM1	-0.2339216	0.00621601	DOWN
STEAP3	-0.2565873	0.00054847	DOWN
STC1	-0.2203364	0.00438777	DOWN
ST3GAL6	-0.2166892	0.00854701	DOWN
ST13	0.20070598	0.00438777	up
SSTR4	-0.2642304	0.00205677	DOWN
SSTR2	-0.3058382	0.00621601	DOWN
SRPK1	-0.2162109	0.00854701	DOWN
SRC	0.21140941	0.00438777	up
SPIB	-0.2576385	0.00205677	DOWN
SPATA16	-0.2332154	0.00438777	DOWN
SP3	-0.2207487	0.0030623	DOWN
SOX4	0.25868479	0.00438777	up

SOGA2	0.21052934	0.00854701	up
SNORD64	-0.2180354	0.00854701	DOWN
SNORD44	-0.3012842	0.00137118	DOWN
SNORA15	-0.2386127	0.00854701	DOWN
SNORA14A	-0.2132989	0.00854701	DOWN
SNCA	0.30566773	0.0030623	up
SMPDL3A	-0.2102562	0.00205677	DOWN
SMARCC1	-0.2564809	0.00854701	DOWN
SLC9A8	-0.251514	0.00621601	DOWN
SLC9A6	-0.2041901	0.00854701	DOWN
SLC38A9	-0.2050233	0.0030623	DOWN
SLC22A5	0.22806799	0.00621601	up
SLC16A3	0.27874145	0.00438777	up
SIPA1L3	0.28440135	0.00854701	up
SIK3-IT1	-0.219126	0.00018282	DOWN
SIGLEC12	0.26501978	0.00438777	up
SHROOM2	0.27207262	0.00438777	up
SHKBP1	0.232133	0.0030623	up
SH3YL1	-0.2406196	0.00854701	DOWN
SGSM1	-0.2239042	0.00854701	DOWN
SFRP5	0.32221175	0.00621601	up
SERPINI1	-0.2438379	0.00854701	DOWN
SERPINE3	-0.2402039	0.00854701	DOWN
SERF2	-0.2082185	0.00621601	DOWN
SEPN1	0.25683502	0.00854701	up
SEMA4D	-0.2293805	0.00137118	DOWN
SECTM1	0.21570416	0.00854701	up
SCPEP1	-0.2231957	0.0030623	DOWN
SALL3	-0.2271562	0.00621601	DOWN
SALL1	0.24815043	0.00854701	up
RTBDN	0.30448347	0.00205677	up
RRM2B	-0.2252645	0.00438777	DOWN
RPS8	-0.2085649	0.0030623	DOWN
RPL35	-0.2035248	0.00086841	DOWN
RPH3AL	0.3136723	0.00086841	up
RPE65	-0.20575	0.0030623	DOWN
RP6-1O2.1	-0.2495813	0.00621601	DOWN
RP5-984P4.4	-0.2729149	0.00438777	DOWN
RP5-916L7.1	-0.243506	0.00438777	DOWN
RP5-865N13.2	-0.2342663	0.00438777	DOWN
RP5-862P8.2	-0.2867907	0.00854701	DOWN
RP5-842K16.1	-0.261781	0.00621601	DOWN
RP5-1166F10.1	-0.2244076	0.00438777	DOWN
RP5-1121H13.4	-0.2173698	0.00621601	DOWN

RP5-1100I6.2	-0.2432223	0.00621601	DOWN
RP5-1024G6.2	-0.2133486	0.00438777	DOWN
RP5-1006K12.1	-0.2021643	0.00854701	DOWN
RP4-745E8.2	0.20134681	0.00854701	up
RP4-695O20__B.10	-0.2425386	0.00621601	DOWN
RP4-673D20.3	0.26901773	0.00086841	up
RP4-614N24.1	-0.2463915	0.00854701	DOWN
RP3-448I9.2	-0.2444265	0.00621601	DOWN
RP1-90G24.10	-0.2167004	0.00438777	DOWN
RP1-84O15.2	-0.2421939	0.00205677	DOWN
RP1-67K17.3	-0.21734	0.00854701	DOWN
RP13-884E18.4	-0.2239247	0.00438777	DOWN
RP1-30G7.2	-0.2249181	0.00854701	DOWN
RP1-292B18.4	-0.2362929	0.00854701	DOWN
RP1-273G13.3	-0.2493796	0.00205677	DOWN
RP1-251I12.1	-0.2001991	0.00438777	DOWN
RP1-213J1P__B.2	-0.2217283	0.0030623	DOWN
RP11-98J9.3	-0.2175257	0.00137118	DOWN
RP11-960D24.1	-0.2447573	0.00205677	DOWN
RP11-933H2.4	-0.2796536	0.00621601	DOWN
RP1-192P9.1	-0.2040022	0.00137118	DOWN
RP11-88H9.1	-0.2106845	0.00854701	DOWN
RP11-863K10.4	-0.2177162	0.00854701	DOWN
RP11-834C11.3	-0.2072936	0.00438777	DOWN
RP11-79P5.5	-0.2078674	0.00438777	DOWN
RP11-78H24.1	-0.2023201	0.0030623	DOWN
RP11-787D18.1	-0.214919	0.00854701	DOWN
RP11-779O18.3	-0.2077956	0.00854701	DOWN
RP11-76G10.1	-0.2027246	0.00854701	DOWN
RP11-767N15.1	-0.2110582	0.00854701	DOWN
RP11-755E23.3	-0.2020129	0.00854701	DOWN
RP11-753G20.1	-0.201246	0.00621601	DOWN
RP11-736K20.5	-0.2190805	0.00621601	DOWN
RP11-735G4.1	-0.2058861	0.00854701	DOWN
RP11-733O18.1	-0.2083746	0.0030623	DOWN
RP11-722M1.1	0.24998724	0.00018282	up
RP11-718G2.4	-0.2091518	0.00854701	DOWN
RP11-716H6.2	-0.2257448	0.00205677	DOWN
RP11-716D16.1	-0.2215672	0.00137118	DOWN
RP11-713M15.1	-0.2853781	0.00137118	DOWN
RP11-711M9.1	-0.2111925	0.00854701	DOWN
RP11-711G10.1	-0.2173098	0.00854701	DOWN
RP11-708B6.2	-0.237795	0.00205677	DOWN
RP11-683L23.4	-0.2413351	0.00621601	DOWN

RP11-680F8.1	-0.2591005	0.00086841	DOWN
RP11-675F6.4	-0.2132539	0.00137118	DOWN
RP11-661I21.2	-0.2044283	0.00137118	DOWN
RP11-65D24.2	-0.3082331	0.00854701	DOWN
RP11-654A16.1	0.26586234	0.00137118	up
RP11-648L3.2	-0.2212572	0.00621601	DOWN
RP11-63K6.7	-0.2112985	0.0030623	DOWN
RP11-631B21.2	-0.2790935	0.00438777	DOWN
RP11-597A11.1	-0.2565174	0.00086841	DOWN
RP11-57H12.5	-0.204909	0.00205677	DOWN
RP11-57A19.2	-0.2043392	0.00621601	DOWN
RP11-568A7.2	-0.2255328	0.00205677	DOWN
RP11-545I5.3	-0.2144498	0.00438777	DOWN
RP11-542G1.2	-0.2265892	0.00438777	DOWN
RP11-517B11.4	-0.266668	0.0030623	DOWN
RP11-508M1.3	0.25366287	0.00205677	up
RP11-503N18.2	-0.2254782	0.0030623	DOWN
RP11-503C24.1	-0.2347906	0.00018282	DOWN
RP11-500G10.1	-0.2674168	0.00854701	DOWN
RP11-49G10.3	-0.2671512	0.00438777	DOWN
RP11-498J9.2	-0.2176009	0.00137118	DOWN
RP11-486M23.2	-0.225063	0.00854701	DOWN
RP11-482D24.3	-0.2184128	0.00621601	DOWN
RP11-468I15.1	-0.2100622	0.0030623	DOWN
RP11-463J7.2	-0.207085	0.00621601	DOWN
RP11-453E2.2	-0.2464229	0.0030623	DOWN
RP11-44M6.1	-0.2029957	0.0030623	DOWN
RP11-44D19.1	-0.2146899	0.00854701	DOWN
RP11-446F3.2	-0.2092463	0.00854701	DOWN
RP11-445N18.7	-0.2432203	0.00621601	DOWN
RP11-444D3.1	0.2223876	0.0030623	up
RP11-439E19.8	-0.2071108	0.00205677	DOWN
RP11-432N13.4	-0.2516643	0.00205677	DOWN
RP1-142L7.5	-0.2205565	0.00137118	DOWN
RP11-429H9.4	-0.2259902	0.00854701	DOWN
RP11-421L21.3	-0.2225877	0.00854701	DOWN
RP11-420J11.2	-0.2101415	0.00621601	DOWN
RP11-415I12.3	-0.2001826	0.00205677	DOWN
RP11-415C15.2	-0.2185754	0.00621601	DOWN
RP11-40H20.4	0.20173039	0.00621601	up
RP11-404P21.6	-0.200107	0.00854701	DOWN
RP11-3J1.1	0.23391424	0.0030623	up
RP11-3D4.2	-0.2186034	0.00438777	DOWN
RP11-398F12.1	-0.2028519	0.00438777	DOWN

RP11-394I13.1	0.23201499	0.00621601	up
RP11-394B5.2	-0.2026699	0.00854701	DOWN
RP11-392P7.8	-0.239724	0.00054847	DOWN
RP11-383C5.7	-0.2659259	0.00137118	DOWN
RP11-382F24.2	-0.2167783	0.0030623	DOWN
RP11-373E16.3	-0.2085183	0.00854701	DOWN
RP11-361D14.2	-0.2204158	0.00854701	DOWN
RP11-356K23.1	-0.2298878	0.00621601	DOWN
RP11-355I22.7	-0.2107934	0.00854701	DOWN
RP11-354P17.15	-0.2271306	0.00621601	DOWN
RP11-340I6.3	0.25308939	0.00854701	up
RP11-336K24.4	-0.2144517	0.00438777	DOWN
RP11-332E19.2	-0.250362	0.00438777	DOWN
RP11-330L19.4	-0.2046513	0.00621601	DOWN
RP11-32B5.7	-0.2280443	0.00438777	DOWN
RP11-321G12.1	-0.2306459	0.00854701	DOWN
RP11-315H15.1	-0.2082701	0.00438777	DOWN
RP11-313A24.1	-0.230158	0.0030623	DOWN
RP11-304C12.3	-0.222977	0.00621601	DOWN
RP11-2O17.2	-0.2107565	0.0030623	DOWN
RP11-2L4.1	-0.2623721	0.00621601	DOWN
RP11-2C15.1	-0.2252996	0.00438777	DOWN
RP11-298D21.1	-0.2253276	0.00854701	DOWN
RP11-281H11.1	-0.2406256	0.00854701	DOWN
RP11-26M5.2	-0.247614	0.00621601	DOWN
RP11-267D19.1	-0.2462526	0.00854701	DOWN
RP11-266O8.1	0.20294205	0.00621601	up
RP11-266E14.1	-0.227889	0.00205677	DOWN
RP11-265D19.6	-0.2425769	0.00621601	DOWN
RP11-261C10.3	-0.2002512	0.00054847	DOWN
RP11-25C19.3	-0.2181371	0.00205677	DOWN
RP11-25C19.1	0.32617842	0.00137118	up
RP11-258F22.1	-0.2195559	0.0030623	DOWN
RP11-257K9.7	-0.2180082	0.00621601	DOWN
RP11-255H23.2	-0.2253267	0.00621601	DOWN
RP11-245M24.1	-0.2333514	0.00438777	DOWN
RP11-240M16.1	0.20679495	0.00205677	up
RP11-23J18.1	-0.2213952	0.00205677	DOWN
RP11-232L2.2	-0.2063994	0.0030623	DOWN
RP1-122P22.2	-0.2027698	0.00438777	DOWN
RP11-221N13.4	-0.227807	0.00854701	DOWN
RP11-21C4.1	0.22821493	0.00621601	up
RP11-218E20.6	-0.2120943	0.00621601	DOWN
RP11-20J15.3	-0.204613	0.00438777	DOWN

RP11-203P2.2	-0.2196781	0.00438777	DOWN
RP11-203F10.5	0.22373569	0.00854701	up
RP11-1D12.2	-0.2290998	0.00621601	DOWN
RP11-199O14.1	-0.2709795	0.00205677	DOWN
RP11-185E8.1	-0.2138344	0.00137118	DOWN
RP11-184A2.3	0.20914457	0.0000457	up
RP11-182J1.12	-0.2597194	0.00854701	DOWN
RP11-178G16.2	-0.2436851	0.0030623	DOWN
RP11-171L9.1	-0.2231975	0.00854701	DOWN
RP11-168O10.6	-0.2123781	0.00854701	DOWN
RP11-168G22.3	0.21152756	0.00018282	up
RP11-168G16.2	-0.2430273	0.00854701	DOWN
RP11-168C9.1	-0.2317487	0.00854701	DOWN
RP11-162J8.2	-0.2426726	0.00438777	DOWN
RP11-161M6.4	0.26660462	0.00205677	up
RP11-159N11.3	-0.2120938	0.00438777	DOWN
RP11-159K7.2	-0.2348911	0.00854701	DOWN
RP11-157E14.1	-0.2741539	0.00205677	DOWN
RP11-152L20.3	-0.2319908	0.00854701	DOWN
RP11-146E13.4	-0.2341559	0.00205677	DOWN
RP11-145A3.2	-0.2234117	0.00438777	DOWN
RP11-143J24.1	0.24066218	0.00854701	up
RP11-140A10.3	-0.2072357	0.00621601	DOWN
RP11-13A1.3	0.28458737	0.00054847	up
RP11-138C24.1	-0.2299361	0.00854701	DOWN
RP11-137L10.6	-0.2141814	0.00621601	DOWN
RP11-132M7.3	-0.2179818	0.00854701	DOWN
RP11-131L23.1	-0.2252772	0.00854701	DOWN
RP11-12K6.2	-0.2522816	0.00205677	DOWN
RP11-129B22.2	-0.2061706	0.00854701	DOWN
RP11-125P18.1	-0.2057339	0.00854701	DOWN
RP11-1252I4.2	-0.2176731	0.00854701	DOWN
RP11-118N24.3	-0.213692	0.00438777	DOWN
RP11-114H21.2	-0.2034792	0.00854701	DOWN
RP11-109E10.1	-0.2144536	0.00205677	DOWN
RP11-106M3.3	-0.2132012	0.00621601	DOWN
RP11-104D21.3	-0.2005685	0.00854701	DOWN
RP11-100L22.1	-0.2409608	0.00438777	DOWN
RORB	0.20607495	0.00621601	up
ROPN1	0.24387607	0.00031994	up
RNY4P19	-0.2661345	0.00018282	DOWN
RNY4P1	-0.2006279	0.00438777	DOWN
RNY3P9	-0.2505253	0.00086841	DOWN
RNY1P8	-0.2001681	0.00621601	DOWN

RNU5F-8P	-0.2005443	0.00854701	DOWN
RNase_MRP	-0.2068492	0.00854701	DOWN
RN5S73	-0.23174	0.00205677	DOWN
RN5S389	-0.2471738	0.0030623	DOWN
RN5S314	-0.2524305	0.00086841	DOWN
RN5S305	-0.215478	0.00854701	DOWN
RN5S275	-0.2298857	0.00854701	DOWN
RN5S261	-0.2015565	0.00854701	DOWN
RN5S196	-0.2576097	0.00621601	DOWN
RN5S191	-0.2292111	0.00854701	DOWN
RN5S163	-0.2109043	0.00137118	DOWN
RN5-8S2	-0.2183353	0.00054847	DOWN
RMND1	-0.2146631	0.00621601	DOWN
RIPK3	0.2190276	0.00621601	up
RIN3	-0.2635031	0.00621601	DOWN
RIBC1	-0.2396301	0.00854701	DOWN
RFPL3-AS1	-0.2049587	0.00438777	DOWN
RFC5	-0.2206351	0.00438777	DOWN
RFC2	-0.4120525	0.00137118	DOWN
REEP1	0.22047797	0.0030623	up
RBP2	-0.2616747	0.00621601	DOWN
RBM11	-0.2228564	0.00621601	DOWN
RBFOX3	0.22047502	0.00438777	up
RALGDS	0.23761103	0.00854701	up
RAD51	-0.2449401	0.00137118	DOWN
RAD1	-0.2427786	0.00854701	DOWN
RAB42	-0.2828227	0.00438777	DOWN
RAB25	-0.3241402	0.0000457	DOWN
RAB10	-0.2274416	0.00621601	DOWN
PTPRQ	0.2416316	0.00854701	up
PTPRK	-0.213498	0.00205677	DOWN
PTPN5	0.31371805	0.00854701	up
PTH1R	0.24965969	0.00854701	up
PTGER3	-0.2556616	0.0030623	DOWN
PRUNE2	-0.2254942	0.00854701	DOWN
PRR23A	-0.203972	0.0030623	DOWN
PRR21	0.29962432	0.00031994	up
PRKX-AS1	-0.2167883	0.00854701	DOWN
PRAMEL	-0.2458815	0.00621601	DOWN
PPP1R3G	-0.2357612	0.0030623	DOWN
PPP1R16B	0.23247398	0.0030623	up
POMT2	-0.214764	0.0030623	DOWN
POLQ	-0.2036639	0.00031994	DOWN
PLSCR1	-0.2022931	0.00621601	DOWN

PLEKHG1	-0.2077365	0.00854701	DOWN
PLEKHA5	0.20344956	0.00621601	up
PLEKHA4	-0.2070027	0.00854701	DOWN
PLCXD3	-0.2619097	0.0030623	DOWN
PLCH2	-0.2301363	0.00854701	DOWN
PLA1A	-0.3037898	0.00621601	DOWN
PFKFB3	-0.3456574	0.00854701	DOWN
PDE2A	0.20184743	0.00054847	up
PCSK7	-0.2317781	0.00621601	DOWN
PCK2	-0.2160948	0.00438777	DOWN
PCIF1	0.21611837	0.00086841	up
PCID2	-0.3677404	0.00621601	DOWN
PAX6	0.28179362	0.00621601	up
PARP11	-0.2256011	0.00621601	DOWN
PAQR8	0.2750412	0.00438777	up
PANK2	-0.289886	0.0030623	DOWN
PALM2	-0.2235428	0.0030623	DOWN
PAK6	0.20299352	0.00854701	up
PAK1	0.29467524	0.00854701	up
PAGE4	-0.2283836	0.00205677	DOWN
PAFAH1B2	-0.2066982	0.00621601	DOWN
PADI2	-0.2411812	0.00054847	DOWN
PACS2	0.41568857	0.0000457	up
OTX2	-0.2252443	0.00438777	DOWN
OR9G4	-0.2013096	0.00621601	DOWN
OR7D4	-0.2205257	0.00438777	DOWN
OR5D14	-0.2262538	0.0030623	DOWN
OR5AP2	-0.236592	0.00438777	DOWN
OR5A1	-0.2091409	0.0030623	DOWN
OR51S1	0.22642425	0.00621601	up
OR51L1	-0.2324151	0.00438777	DOWN
OR51E2	-0.2358534	0.00854701	DOWN
OR4S1	0.24733576	0.00621601	up
OR2T33	-0.2164089	0.00137118	DOWN
OR2K2	-0.2632327	0.00137118	DOWN
OR2AP1	-0.2092083	0.00621601	DOWN
OR1L4	-0.2816917	0.0030623	DOWN
OR1B1	-0.217699	0.00854701	DOWN
OR13H1	-0.2303236	0.00854701	DOWN
OR13C3	-0.21004	0.00854701	DOWN
OR10A5	-0.2174429	0.00854701	DOWN
OPTC	-0.2314474	0.00438777	DOWN
OGDH	-0.2102318	0.00854701	DOWN
ODZ2	0.24210438	0.00854701	up

OAS3	-0.2544594	0.00205677	DOWN
OAS1	-0.206193	0.00621601	DOWN
NYAP1	0.23825656	0.00621601	up
NXPH1	0.25644191	0.00854701	up
NUP214	-0.2048081	0.00621601	DOWN
NTS	-0.2297742	0.00205677	DOWN
NTRK3	0.23467209	0.0030623	up
NOTCH3	-0.2674099	0.00438777	DOWN
NME3	-0.2587159	0.00054847	DOWN
NMBR	-0.2482657	0.00205677	DOWN
NLRP3	0.21727846	0.00137118	up
NKAIN4	0.20331725	0.0030623	up
NGB	-0.2046059	0.00438777	DOWN
NEGR1-AS1	-0.2070422	0.00854701	DOWN
NEFL	0.25417846	0.00854701	up
NCR1	0.20883941	0.00086841	up
NCAPD3	-0.2224036	0.00205677	DOWN
NBEAP1	-0.2170929	0.00438777	DOWN
NAT16	0.28633844	0.00438777	up
NAP1L5	0.21082151	0.00854701	up
NALCN-AS1	-0.2280637	0.00621601	DOWN
N4BP2	-0.257158	0.00621601	DOWN
MYL5	0.26877193	0.00205677	up
MYH9	0.23644489	0.00854701	up
MYCT1	-0.2760938	0.00018282	DOWN
MX1	0.22863924	0.00854701	up
MUSTN1	-0.2951455	0.0030623	DOWN
MTUS2-AS2	-0.2083327	0.00438777	DOWN
MTSS1	0.26143135	0.00205677	up
MTRNR2L3	-0.2098941	0.00438777	DOWN
MTCH1	-0.2073139	0.0030623	DOWN
MRPL35	-0.2040608	0.00137118	DOWN
MRGPRX1	-0.2625367	0.00054847	DOWN
MRAP	-0.2028692	0.00054847	DOWN
MOSPD1	-0.2266524	0.00621601	DOWN
MMP3	-0.2609066	0.00621601	DOWN
MIXL1	-0.2141181	0.00137118	DOWN
MIR892B	-0.2507399	0.00205677	DOWN
MIR660	-0.2129718	0.00438777	DOWN
MIR651	0.24926822	0.00137118	up
MIR646	0.25792567	0.00854701	up
MIR502	-0.2001266	0.00086841	DOWN
MIR193B	-0.2052887	0.00854701	DOWN
MIR135A2	-0.2101886	0.00854701	DOWN

MIR1298	-0.2335126	0.00854701	DOWN
MIR1193	-0.2143096	0.00205677	DOWN
MIR1185-2	-0.2227153	0.00205677	DOWN
MIPOL1	-0.2373497	0.0030623	DOWN
MIF4GD	-0.219742	0.00621601	DOWN
MGP	-0.2599835	0.00205677	DOWN
MGAT2	-0.2133822	0.0030623	DOWN
METTL17	-0.2391572	0.00621601	DOWN
MESP1	-0.2042253	0.00205677	DOWN
MERTK	-0.2625844	0.00438777	DOWN
MEIS2	0.23704315	0.00205677	up
MEGF11	0.28542083	0.0030623	up
MEF2B	0.31120487	0.00854701	up
MED24	0.21000758	0.00854701	up
MATK	0.25211547	0.0030623	up
MAST4	0.22589407	0.00137118	up
MAST2	0.22513547	0.00438777	up
MAP3K7	-0.2487408	0.00854701	DOWN
MAML1	-0.2441075	0.00621601	DOWN
MAGI2-AS3	-0.2495374	0.00621601	DOWN
MAGI2-AS1	-0.2003037	0.00854701	DOWN
MAGEA3	-0.205205	0.00205677	DOWN
MACROD1	-0.2180143	0.00054847	DOWN
LYPD6B	0.24520561	0.00086841	up
LYPD4	-0.2265255	0.00438777	DOWN
LUZP1	-0.2111247	0.00854701	DOWN
LRRFIP1	0.34534531	0.00854701	up
LRRC9	-0.2023941	0.00854701	DOWN
LRRC8D	0.25732873	0.00018282	up
LRRC38	0.28998504	0.0030623	up
LRRC31	-0.2537211	0.00621601	DOWN
LNX1-AS1	-0.2608229	0.00621601	DOWN
LMX1A	-0.2220958	0.00621601	DOWN
LMO7	0.23670879	0.00205677	up
LMO2	0.21476634	0.00621601	up
LMBR1L	0.27041659	0.00438777	up
LL22NC03-23C6.13	-0.2950041	0.00031994	DOWN
LL0XNC01-37G1.1	-0.2461211	0.0030623	DOWN
LIPG	0.22853259	0.00854701	up
LINC00502	-0.2423405	0.00137118	DOWN
LINC00481	-0.2122628	0.00621601	DOWN
LINC00467	-0.2152165	0.0030623	DOWN
LINC00316	0.28641454	0.00854701	up
LINC00237	0.26243964	0.00438777	up

LHCGR	-0.2298044	0.00621601	DOWN
LCE4A	-0.2531799	0.0030623	DOWN
L3MBTL3	0.21561721	0.00137118	up
L1CAM	0.2510021	0.00854701	up
KSR2	0.23703898	0.00854701	up
KRTAP5-5	-0.2059116	0.00438777	DOWN
KRTAP19-2	-0.2008292	0.00854701	DOWN
KRT7	-0.2316159	0.00438777	DOWN
KRT38	0.32545154	0.0000914	up
KRT33A	-0.2347833	0.00854701	DOWN
KNG1	-0.208961	0.00854701	DOWN
KLHL36	-0.213082	0.0030623	DOWN
KLHDC2	-0.2128269	0.00621601	DOWN
KIR3DL1	0.26687977	0.00854701	up
KIAA1524	-0.2159194	0.0030623	DOWN
KIAA1467	-0.2296774	0.00086841	DOWN
KIAA1257	-0.2388433	0.0030623	DOWN
KIAA1211	-0.2369597	0.00018282	DOWN
KIAA0226	0.22861091	0.00438777	up
KIAA0020	-0.2029077	0.00854701	DOWN
KCTD16	-0.2304064	0.00854701	DOWN
KCTD15	-0.2161243	0.00438777	DOWN
KCNK10	0.22854076	0.00854701	up
KCNJ6	-0.208059	0.00854701	DOWN
KBTBD3	-0.2211193	0.00438777	DOWN
KB-1043D8.6	-0.2049681	0.00854701	DOWN
KALRN	0.26127657	0.00854701	up
ITPR2	-0.2219786	0.00621601	DOWN
ITPKA	0.24849468	0.00205677	up
ITGB6	-0.2359281	0.00205677	DOWN
ITGB1BP2	-0.2068774	0.00137118	DOWN
ITGB1BP1	-0.2719852	0.00621601	DOWN
ITGAX	0.23968453	0.00137118	up
IL33	-0.211523	0.00854701	DOWN
IL17D	-0.2100254	0.00854701	DOWN
IKZF3	0.23284249	0.00621601	up
IKBKB	-0.2275904	0.00854701	DOWN
IGSF1	-0.231617	0.0030623	DOWN
IGLJ2	-0.2117274	0.0030623	DOWN
IGHD6-13	0.26734337	0.00137118	up
IGF2BP1	-0.2086245	0.00854701	DOWN
IFNA16	-0.207085	0.00854701	DOWN
IDS	-0.2058696	0.00621601	DOWN
ID3	0.25777687	0.00854701	up

ID1	0.24939215	0.00137118	up
HSPB8	-0.2762352	0.00854701	DOWN
HRH1	0.25573739	0.00854701	up
HOXD4	-0.2128853	0.00438777	DOWN
HOGA1	0.20866554	0.00621601	up
HMOX2	-0.3118231	0.0030623	DOWN
HMGA1P1	-0.2390669	0.00205677	DOWN
HLCS	-0.2784145	0.0030623	DOWN
HLA-L	-0.2264166	0.00438777	DOWN
HLA-E	-0.2718436	0.00621601	DOWN
HLA-DQB1	-0.2147833	0.00137118	DOWN
HLA-DPB1	-0.2397978	0.00054847	DOWN
HIVEP3	0.20273975	0.00854701	up
HIST1H2BJ	-0.2171633	0.00854701	DOWN
HHLA2	0.4456068	0.00086841	up
HAS2	-0.2614036	0.00621601	DOWN
GZMK	-0.2082081	0.00086841	DOWN
GSTO1	-0.2062376	0.00854701	DOWN
GS1-542M4.4	-0.2372593	0.00438777	DOWN
GRXCR1	-0.2337924	0.00854701	DOWN
GRIK1-AS2	-0.2169985	0.00086841	DOWN
GRIK1	0.2812778	0.00854701	up
GPR55	0.20247148	0.00621601	up
GPR176	0.24141623	0.00205677	up
GPHB5	-0.2097268	0.00854701	DOWN
GP6	-0.2161759	0.0030623	DOWN
GNG7	0.27409518	0.00018282	up
GNB1L	-0.2285073	0.00438777	DOWN
GNAT2	-0.2106464	0.0030623	DOWN
GNA11	-0.2459341	0.0030623	DOWN
GLRA2	-0.2077319	0.00205677	DOWN
GLRA1	0.29172836	0.00438777	up
GLOD4	-0.2399081	0.00854701	DOWN
GK-AS1	-0.2369508	0.0030623	DOWN
GJB4	0.26224854	0.00621601	up
GFPT2	-0.3356109	0.00137118	DOWN
GCNT2	-0.2609031	0.0030623	DOWN
GATA2	0.20107967	0.00854701	up
GATA1	-0.2362474	0.00854701	DOWN
GAL3ST2	0.21673874	0.00854701	up
GAL	-0.2342322	0.00854701	DOWN
GABBR1	0.22355818	0.00854701	up
G6PD	0.20797984	0.0000457	up
FXYD2	-0.2548982	0.00205677	DOWN

FUNDC1	-0.208468	0.00438777	DOWN
FRMPD4-AS1	-0.237845	0.00621601	DOWN
FOXB1	-0.2357196	0.00854701	DOWN
FGF5	0.37670201	0.00137118	up
FEZ1	0.24501882	0.0030623	up
FDX1	-0.2340293	0.00854701	DOWN
FBXO45	-0.2235555	0.00086841	DOWN
FBN2	0.36188784	0.00621601	up
FANCB	-0.2348976	0.00854701	DOWN
FAM83C	-0.2071293	0.00854701	DOWN
FAM78B	0.22033674	0.00137118	up
FAM47E	0.21102629	0.00205677	up
FAM41C	-0.256299	0.00205677	DOWN
FAM181A	-0.2818284	0.00137118	DOWN
FAM166A	-0.2114796	0.00854701	DOWN
FAM155A-IT1	0.23446252	0.00621601	up
FAM13B	-0.2598729	0.00621601	DOWN
FAM13A	0.20590669	0.00438777	up
FAM114A1	0.24527167	0.00438777	up
EXOC3	0.28096387	0.00438777	up
ESRRG	0.28193992	0.0030623	up
ERLIN2	-0.2326831	0.00854701	DOWN
ERI1	-0.2149366	0.00621601	DOWN
ERCC5	-0.2047419	0.00854701	DOWN
ERAS	-0.2188176	0.00621601	DOWN
EPHA5	0.24631884	0.00621601	up
EPHA2	0.21428456	0.00854701	up
ENTPD4	0.20511702	0.00854701	up
ELMOD1	-0.2202427	0.00438777	DOWN
ELMO1	0.30588763	0.00018282	up
EIF2C1	0.21751631	0.0030623	up
EIF2AK1	-0.3295555	0.00438777	DOWN
EGFEM1P	0.24210072	0.00621601	up
DYNC2LI1	-0.272338	0.0030623	DOWN
DUSP5	-0.2243451	0.00438777	DOWN
DSCR4	-0.2438532	0.00205677	DOWN
DPH2	-0.2230926	0.00854701	DOWN
DPF1	0.30626274	0.00086841	up
DOK4	0.22674834	0.00854701	up
DNTT	-0.2313608	0.00621601	DOWN
DLX6	0.2048722	0.00854701	up
DLGAP2	0.37992983	0.00854701	up
DLG2	0.27295538	0.00854701	up
DKKL1	-0.2097093	0.00205677	DOWN

DIAPH2	-0.2013605	0.00621601	DOWN
DIAPH1	-0.2060119	0.0030623	DOWN
DGKE	-0.2480173	0.00854701	DOWN
DEPDC4	-0.2110132	0.00621601	DOWN
DENND1C	-0.2002213	0.00438777	DOWN
DEFB119	-0.2385238	0.00621601	DOWN
DDX39B	-0.2267603	0.00137118	DOWN
DDX18P6	-0.2228527	0.00621601	DOWN
DACH1	0.23845744	0.00137118	up
CYTH1	0.24610402	0.00438777	up
CYP7B1	-0.2238934	0.00621601	DOWN
CYP3A43	-0.2093363	0.00621601	DOWN
CUTC	-0.2060999	0.00621601	DOWN
CUL4B	-0.2123439	0.00854701	DOWN
CTNNA2	0.32074161	0.00086841	up
CTD-3253I12.1	-0.2727229	0.00854701	DOWN
CTD-2647L4.1	-0.3104421	0.00438777	DOWN
CTD-2574D22.1	-0.2308291	0.00854701	DOWN
CTD-2566J3.1	-0.2031823	0.00205677	DOWN
CTD-2555A7.2	0.22583759	0.00621601	up
CTD-2534J5.1	-0.2514721	0.00854701	DOWN
CTD-2331D11.3	0.26076725	0.0030623	up
CTD-2314I6.1	-0.2133498	0.00854701	DOWN
CTD-2139B15.4	-0.2434435	0.00205677	DOWN
CTD-2134A5.4	0.22371042	0.00137118	up
CTD-2116N24.1	-0.2641026	0.00438777	DOWN
CTD-2050B12.2	0.23856448	0.00854701	up
CTD-2014E2.5	-0.2304462	0.00205677	DOWN
CTCFL	-0.2398464	0.00621601	DOWN
CTC-436P18.3	0.21487013	0.00621601	up
CTC-261N6.1	-0.2275015	0.00018282	DOWN
CTC-228N24.1	-0.2319749	0.00205677	DOWN
CTB-32P11.1	-0.2278706	0.00086841	DOWN
CTB-27N1.1	-0.21256	0.00205677	DOWN
CTB-26E19.1	-0.2170171	0.00438777	DOWN
CTAGE5	-0.2263296	0.00621601	DOWN
CTA-929C8.7	-0.2504396	0.00086841	DOWN
CTA-292E10.6	-0.2177574	0.00621601	DOWN
CREB3L4	-0.2555134	0.00054847	DOWN
CPO	-0.3673615	0.00205677	DOWN
CPA6	0.20979485	0.00205677	up
CPA2	0.36228808	0.00621601	up
COPS5	0.2397254	0.00621601	up
COL11A2	0.21125881	0.00086841	up

CNTN4	0.29827686	0.00205677	up
CNPY1	-0.2466661	0.00438777	DOWN
CNFN	0.20376841	0.00854701	up
CMIP	0.21831836	0.00854701	up
CLNS1A	-0.2476922	0.00438777	DOWN
CLEC9A	-0.2694848	0.00086841	DOWN
CIB4	-0.2158023	0.00205677	DOWN
CGB5	-0.2502501	0.0030623	DOWN
CERS4	-0.229608	0.00854701	DOWN
CEP170P1	-0.2449342	0.00621601	DOWN
CEP128	0.24890363	0.00031994	up
CDRT15L2	-0.2193172	0.00621601	DOWN
CDH23	0.20602095	0.00854701	up
CD82	0.22555385	0.0000457	up
CD247	0.21844491	0.00205677	up
CD200R1	-0.2152914	0.00621601	DOWN
CD200	0.26253331	0.00205677	up
CD1E	0.22950439	0.00854701	up
CD177	0.28958611	0.00621601	up
CD109	0.22382702	0.00137118	up
CCNY	0.26549672	0.00086841	up
CCDC88A	0.20080345	0.00438777	up
CCDC72	-0.2104955	0.00854701	DOWN
CCDC56	-0.2464744	0.00621601	DOWN
CCDC48	0.21081456	0.0030623	up
CCDC38	0.22781625	0.00621601	up
CCDC130	-0.2401226	0.00621601	DOWN
CCDC129	0.20643927	0.00854701	up
CCDC101	-0.2036431	0.00854701	DOWN
CASZ1	0.24225176	0.0030623	up
CASP8	0.20085858	0.00621601	up
CASC1	-0.2270796	0.00854701	DOWN
CARM1P1	-0.2262949	0.00621601	DOWN
CARD18	0.27067653	0.0030623	up
CAND2	-0.2776934	0.00621601	DOWN
CAMKV	-0.2086838	0.00205677	DOWN
CACNB2	0.21075692	0.00854701	up
CACNA2D4	0.2292638	0.00018282	up
CABP7	-0.2320532	0.00621601	DOWN
C9orf86	-0.2082618	0.0030623	DOWN
C9orf46	-0.2181304	0.00205677	DOWN
C9orf152	-0.2147442	0.00854701	DOWN
C6orf141	-0.2100066	0.00854701	DOWN
C3orf55	0.2562381	0.00438777	up

C3orf27	0.26132854	0.00086841	up
C3orf14	-0.2163205	0.00621601	DOWN
C2orf71	-0.2187499	0.00854701	DOWN
C2orf40	-0.2274241	0.00621601	DOWN
C1orf61	0.21648973	0.00621601	up
C1orf38	0.20016189	0.00854701	up
C1orf227	-0.2150754	0.00854701	DOWN
C1orf220	-0.2047476	0.0000914	DOWN
C1orf187	-0.2240517	0.00854701	DOWN
C1orf145	0.25910061	0.00854701	up
C1orf124	-0.2176653	0.00854701	DOWN
C1orf106	-0.2349306	0.00854701	DOWN
C18orf63	-0.2565981	0.00854701	DOWN
C16orf78	-0.2403608	0.00854701	DOWN
C15orf55	-0.2006936	0.00621601	DOWN
C14orf43	0.24154502	0.00086841	up
C11orf85	-0.2705999	0.00854701	DOWN
C11orf58	-0.2081828	0.00031994	DOWN
C11orf44	-0.2769752	0.00205677	DOWN
C10orf76	-0.2091134	0.00854701	DOWN
BUB1B	-0.2050645	0.00854701	DOWN
BRWD1	0.21630252	0.00854701	up
BPESC1	-0.2757936	0.00438777	DOWN
BLM	-0.2085445	0.00854701	DOWN
BIRC7	-0.2172473	0.00438777	DOWN
BEST2	0.25121211	0.00438777	up
BDKRB2	0.20607006	0.00086841	up
BCKDK	-0.2009743	0.0030623	DOWN
BACE1	-0.2004691	0.00854701	DOWN
B4GALNT1	0.2091773	0.00854701	up
ATPAF1-AS1	-0.2198924	0.00621601	DOWN
ATP6V1G3	-0.2259113	0.00621601	DOWN
ATP11B	0.22270572	0.00854701	up
ATG13	-0.2350225	0.00137118	DOWN
ATF7IP2	0.24154519	0.00205677	up
ASS1	-0.2014487	0.00854701	DOWN
ASAH2B	0.23965856	0.00621601	up
ARX	-0.2341698	0.00621601	DOWN
ARHGEF38	-0.2431638	0.0030623	DOWN
AQP4	-0.2346617	0.00854701	DOWN
APOA1	-0.2480997	0.00621601	DOWN
APLP1	-0.2083682	0.00205677	DOWN
AP1M2	-0.2464874	0.00854701	DOWN
AP000797.3	-0.2556771	0.00205677	DOWN

AP000704.5	0.20252822	0.0030623	up
AP000688.15	-0.2278747	0.00438777	DOWN
AP000525.1	-0.2257074	0.00438777	DOWN
AP000233.4	-0.2025795	0.00621601	DOWN
AP000233.3	-0.217664	0.00438777	DOWN
ANO7	-0.3078331	0.00854701	DOWN
ANKS6	-0.2172005	0.00854701	DOWN
ANGPTL5	-0.263862	0.00438777	DOWN
AMIGO1	0.24383401	0.00621601	up
AMBRA1	0.26070137	0.00018282	up
ALPP	-0.2744933	0.00854701	DOWN
ALPL	-0.2300461	0.00854701	DOWN
AL589986.2	-0.2127681	0.0030623	DOWN
AL391416.1	-0.2188898	0.0030623	DOWN
AL355512.1	-0.2026226	0.00137118	DOWN
AL109763.2	-0.2048603	0.00205677	DOWN
AL035696.1	-0.2578895	0.00621601	DOWN
AL035088.1	-0.235271	0.00854701	DOWN
AL022344.7	0.30449259	0.0030623	up
AL022344.5	-0.2242842	0.00854701	DOWN
AIPL1	-0.210269	0.00854701	DOWN
AHNAK	-0.2189903	0.00438777	DOWN
AGBL3	-0.2164948	0.00854701	DOWN
AGAP1-IT1	-0.2408418	0.00854701	DOWN
AFF2-IT1	-0.2060459	0.00854701	DOWN
AF186192.2	-0.2228074	0.00854701	DOWN
AF131215.6	0.20715544	0.00854701	up
AF127936.5	-0.2131436	0.00438777	DOWN
AF067845.1	-0.2214705	0.00438777	DOWN
ADCY2	0.31540834	0.00621601	up
ADAM28	0.21362679	0.00086841	up
ACOT13	-0.2587986	0.00205677	DOWN
AC147651.3	0.21981583	0.00621601	up
AC141930.2	-0.2214198	0.00438777	DOWN
AC136188.1	-0.241268	0.00137118	DOWN
AC133633.1	-0.2208967	0.00137118	DOWN
AC121332.1	-0.2696273	0.00438777	DOWN
AC116035.1	-0.2417948	0.0030623	DOWN
AC114877.1	-0.2173793	0.00438777	DOWN
AC114776.3	-0.2349572	0.00621601	DOWN
AC114499.1	-0.2420656	0.00621601	DOWN
AC113607.3	-0.22375	0.00621601	DOWN
AC113331.9	-0.2199551	0.00854701	DOWN
AC108462.1	-0.261019	0.00137118	DOWN

AC108142.1	-0.2097124	0.00438777	DOWN
AC106876.2	-0.2110445	0.00621601	DOWN
AC104417.1	-0.2387587	0.00621601	DOWN
AC104389.16	-0.2370643	0.00438777	DOWN
AC104236.1	-0.2308325	0.00438777	DOWN
AC104012.1	-0.2257926	0.00438777	DOWN
AC103702.1	-0.2485048	0.00621601	DOWN
AC103564.7	-0.2083489	0.00137118	DOWN
AC098617.2	0.3122599	0.00137118	up
AC097468.7	-0.258355	0.00031994	DOWN
AC093693.2	-0.2504989	0.00621601	DOWN
AC093627.8	-0.2930949	0.00086841	DOWN
AC092431.1	-0.2857665	0.0030623	DOWN
AC091705.1	-0.2276258	0.00438777	DOWN
AC091096.1	-0.3342981	0.00086841	DOWN
AC090666.1	-0.2202402	0.00854701	DOWN
AC087433.1	-0.2242671	0.00438777	DOWN
AC087289.1	-0.2452413	0.00086841	DOWN
AC087073.1	0.3041569	0.0030623	up
AC078953.1	-0.264501	0.00205677	DOWN
AC073321.4	-0.2012323	0.00621601	DOWN
AC073321.3	-0.2637099	0.00205677	DOWN
AC073133.1	-0.2257767	0.00854701	DOWN
AC069368.3	-0.2188203	0.00438777	DOWN
AC068535.2	-0.2164625	0.00031994	DOWN
AC024569.1	-0.2005656	0.00438777	DOWN
AC013448.1	-0.2559075	0.00031994	DOWN
AC012531.25	-0.2220491	0.00137118	DOWN
AC012358.8	-0.2233831	0.00854701	DOWN
AC011899.9	0.23454892	0.00854701	up
AC011741.1	-0.2096747	0.00621601	DOWN
AC011306.2	-0.2483436	0.00137118	DOWN
AC009502.3	-0.2352005	0.00621601	DOWN
AC008063.3	0.2414675	0.00438777	up
AC007486.1	-0.2333793	0.00438777	DOWN
AC006380.1	-0.212371	0.00621601	DOWN
AC006372.1	-0.2468738	0.00854701	DOWN
AC005152.2	0.22865709	0.00621601	up
AC005009.1	-0.2204277	0.0030623	DOWN
AC004980.7	-0.2455417	0.00621601	DOWN
AC004869.2	-0.2147286	0.00854701	DOWN
AC004790.1	-0.2424876	0.00621601	DOWN
AC004160.4	-0.2388378	0.00054847	DOWN
AC004112.4	-0.2018981	0.00621601	DOWN

AC003658.1	-0.2037431	0.00854701	DOWN
ABCC5-AS1	-0.437083	0.0000914	DOWN
ABCB11	-0.236564	0.00621601	DOWN
ABCA13	-0.233239	0.00854701	DOWN
A4GALT	0.28417086	0.00205677	up
